# The Parental Pesticide and Offspring’s Epigenome Study: Towards an Integrated Use of Human Biomonitoring of Exposure and Effect Biomarkers

**DOI:** 10.3390/toxics9120332

**Published:** 2021-12-02

**Authors:** Aziza Menouni, Radu Corneliu Duca, Imane Berni, Mohamed Khouchoua, Manosij Ghosh, Brahim El Ghazi, Noura Zouine, Ilham Lhilali, Dina Akroute, Sara Pauwels, Matteo Creta, Katrien Poels, Peter Hoet, Jeroen Vanoirbeeck, Marie-Paule Kestemont, Paul Janssen, Tara Sabo Attwood, Lode Godderis, Samir El Jaafari

**Affiliations:** 1 Cluster of Competence Environment and Health, Moulay Ismail University, Meknes 50000, Morocco; imane.berni@gmail.com (I.B.); khouchouamohamed@gmail.com (M.K.); elghazi.ibrahim@gmail.com (B.E.G.); nora.zouine@edu.umi.ac.ma (N.Z.); ilham.lhilali@edu.umi.ac.ma (I.L.); dina.akroute@edu.umi.ac.ma (D.A.); s.eljaafari@umi.ac.ma (S.E.J.); 2 Health and Environment Unit, Faculty of Medicine, KU Leuven, 3000 Leuven, Belgium; radu.duca@lns.etat.lu (R.C.D.); manosij.ghosh@kuleuven.be (M.G.); sara.pauwels@kuleuven.be (S.P.); matteo.creta@kuleuven.be (M.C.); katrien.poels@kuleuven.be (K.P.); peter.hoet@kuleuven.be (P.H.); jeroen.vanoirbeek@kuleuven.be (J.V.); lode.godderis@kuleuven.be (L.G.); 3 Unit of Environmental Hygiene and Biological Monitoring, Department of Health Protection, Laboratoire National de Santé (LNS), L-3555 Dudelange, Luxembourg; 4 Louvain School of Management, Université Catholique de Louvain, 1348 Louvain-La-Neuve, Belgium; marie-paule.kestemont@uclouvain.be; 5 Center for Statistics, Hasselt University, 3590 Hasselt, Belgium; paul.janssen@uhasselt.be; 6 Department of Environmental and Global Health, University of Florida, Gainesville, FL 32611, USA; sabo@phhp.ufl.edu; 7 IDEWE, External Service for Prevention and Protection at Work, 3001 Heverlee, Belgium

**Keywords:** biomonitoring, pesticides, epigenetic biomarkers, oxidative stress, Morocco

## Abstract

In Morocco, due to the lack of education and the presence of a counterfeit market, pesticides constitute a major problem to be addressed by occupational and environmental health agencies. This paper aims to introduce the PaPOE (Parental Pesticides and Offspring Epigenome) prospective study and its goals, to motivate the study rationale and design, and to examine comprehensively whether multi-residue exposure to commonly used pesticides could induce epigenetic alterations through the oxidative stress pathway. The PaPOE project includes a cross-sectional study assessing the occupational exposure among 300 farmworkers in Meknes, and initiates a birth cohort of 1000 pregnant women. Data and biological samples are collected among farmworkers, and throughout pregnancy, and at birth. Oxidative stress biomarkers include Glutathione, Malondialdehyde, and 8-OHdG. Global and gene-specific DNA methylation is assessed. The study began enrollment in 2019 and is ongoing. As of 30 June 2021, 300 farmworkers and 125 pregnant women have enrolled. The results are expected to showcase the importance of biomonitoring for understanding individual risks, and to identify a number of regions where DNA methylation status is altered in the pesticides-exposed population, paving the way for an integrated biomonitoring system in Morocco and Africa to assess environmental exposures and their long-term health consequences.

## 1. Introduction

According to the Food and Agriculture Organization (FAO), a pesticide is defined as a chemical or biological agent or mixture of agents used for the prevention, control, or extermination of pests. Before reaching the market, pesticides are subjected to toxicity and carcinogenicity tests. Despite these regulatory policies, pesticide exposure continues to be associated with various cancers. In fact, several epidemiological studies have investigated the relation between pesticide exposure and the incidence of cancer [1,2,3,4,5,6,7]. Limited by inadequate or retrospective exposure information, epidemiological studies have been abandoned in favor of a human biomonitoring approach [8]. Subsequently, a growing body of evidence shows that some pesticides might be considered as carcinogens in occupational [9], para-occupational, and/or domestic environments, and are associated with an increased incidence of breast cancer [4,10,11], prostate cancer [3,12,13], bladder cancer [5,10], Non-Hodgkin Lymphoma [11], and even childhood tumors [12,13,14,15]. This increased risk of cancer following pesticides exposure calls for a more focused effort to understand the mechanisms involved, in order to expand the current knowledge of pesticides carcinogenicity and for better hazard identification.

Several classes of pesticides, including organophosphates (OPs), have been shown to interfere with normal system functions, leading to an induction of DNA damage and a decrease in the level of antioxidant enzymes [16,17]. Organochlorines (OCs), with their long half-lives, possess estrogenic properties that contribute to their carcinogenic effects, especially in breast tissue [18]. Pyrethroid pesticides exposure, as well as exposure to other neurotoxicants, are associated with neurodegenerative diseases because they can interfere with mitochondrial function and increase oxidative stress and alpha-synuclein aggregation by genetic and epigenetic mechanisms. Attempts on animal models and human cells in vitro have tried to explain the mechanism by which pesticides interfere with normal body functions. Endosulfan and Dieldrin have been suggested to increase cell proliferation in vitro [19], Organochlorines were associated with a decrease in glutathione-S-transferase (GST) and an increase in glutathione (GSH), leading to an increase in reactive oxygen species (ROS) and generating oxidative damage in the body. The damage that is thus sustained contributes to all stages of the cancer process [20]. In humans, however, the biological mechanisms that could explain the effect of pesticide exposure on cancer incidence and pregnancy outcomes are currently poorly clarified. Novel knowledge linked epigenetic changes to the etiology of chemical-induced carcinogenesis, and epigenetics are, today, known to cooperate with genetic alterations to drive the cancer phenotype [21,22]. Epigenetics is defined as the study of modifications that can occur at a genomic level, leading to a change in the gene expression without any change in the DNA sequence [23,24,25,26]. In other words, the epigenome has the ability to regulate gene expression, leading to distinct phenotypes. Epigenetic programming can, thus, respond differently to environmental stimuli, making it more plastic than the genome [27]. However, epigenetic modifications in animals have been shown to be persistent across generations with a potential link to the fetal programming of later-life health [28]. This explains the fact that environmental exposure to pesticides during critical windows of development can lead to subtle changes in gene expression.

In the same line, it has become more likely that chronic diseases such as cancer have an in utero origin, while early events, including nutrition and environmental exposure during gestation, or even in the pre-conception period, could be harmful not only to parental health but also to offspring, by increasing the susceptibility of the newborn to chronic diseases in adulthood [29,30,31]. This was initially hypothesized by David Barker in 1995, who suggested the pivotal role of the prenatal environment and the postnatal life in the early developmental origin of adult-onset diseases [32], a theory later supported by epigenetics [33]. Likewise, the heritable transmission of environmentally induced phenotypes is referred to as epigenetic trans-generational inheritance. This phenomenon operates during specific critical windows of exposure that are linked to the developmental biology of the germ cells. From this angle, the “Developmental Origins of Health and Disease” hypothesis (DOHaD) describes how the interplay between parental and environmental factors in early life can program fetal development. Similarly, recent evidence suggests that prenatal exposure to diverse environmental chemicals dysregulates the fetal epigenome, with potential consequences for subsequent developmental disorders and diseases manifesting in childhood, over the life-course, or even trans-generationally [31]. Interestingly, it is suggested that all epigenetic modifications, including histone modification, small RNA, and DNA methylation, are all reset following the germline reprogramming, hence interrupting the trans-generational epigenetic inheritance [29]. Returning to pesticides, it has been suggested that long-term ambient OP exposure was associated with differential methylation at 70 CpG sites [34]. In an Agricultural Health study, it has been reported that high pesticides exposure events may increase DNA methylation at specific genes, including GSTp1, for which inactivation has been associated with cancer [35].

According to the ONSSA (National Food Safety Office) in Morocco, there are 1055 commercial pesticides and 375 active compounds [36]. The import value increased at an average annual rate of 10.16%. Even if the import value of some pesticides has decreased (such as DDT, HCB, HCH, Lindane, etc.) [37], counterfeited pesticides and illegal markets continue to be a growing challenge in Morocco. In Meknes, a retrospective inquiry conducted among farmers has revealed a significant use of pesticides (79.3% of farmers use chemical products) [38,39]. In Morocco, non-communicable diseases (NCD) account for 84% of all deaths [40]. Cancer represents the second-highest cause of death, with 18.88% of all deaths and a prevalence of 3.2% among children. Women represented a significantly high proportion of cancer patients. The five most common types of cancers diagnosed were breast (38.2%), cervix (11.4%), colon-rectum (6.7%), bronchus and lung (5.5%), and prostate (4.9%) [41].

Given the above-described situation, the Parental Pesticides and Offspring Epigenome study (PaPOE) was initiated to advance the development of more effective screening methods of pesticides exposure, which form the basis for the future development of standardized prevention and treatment programs. To date, there have been few attempts at establishing longitudinal cohorts in Morocco. Our current study, the PaPOE study, is the first dedicated occupational and longitudinal birth cohort study on pesticides exposure in low-/middle-income countries. It was established to allow for the investigation of factors contributing to epigenetic variation in humans with a link to pesticides exposure. Launched in March 2018, PaPOE was designed as a prospective cohort study to investigate whether pesticides exposure could affect epigenetic changes and increase cancer risk. We will be observing how programming can have long-term consequences for later health outcomes through epigenetic mechanisms, establishing a link between parental pesticides exposure, trans-generational effects, and the health risks for offspring.

We here present the aims and protocols of the PaPOE study and provide a brief overview of the current shortcomings.

### 1.1. Aims of PaPOE

The PaPOE study aims to investigate whether epigenetic changes could be associated with pesticides exposure. Specific objectives are:

- to determine individual exposure to pesticides using biomonitoring in an occupational setting and study the effects on DNA methylation among farmers;
- to assess whether pesticides exposure could affect the occurrence of cancer through the oxidative stress pathway;
- to study the association between maternal exposure to pesticides during pregnancy and DNA methylation pattern in genes related to the oxidative stress pathway;
- to determine if parental pesticides exposure can affect global and gene specific DNA methylation patterns in offspring.

#### 1.1.1. Biomonitoring Pesticides in an Occupational Setting and Determining Their Effects on DNA Methylation among Farmers, Assessing the Occurrence of Cancer through the Oxidative Stress Pathway

It is well-established that relevant exposure levels of pesticides can lead to a variety of adverse health outcomes. Global DNA hypomethylation has been reported in human studies and was correlated with a high level of pesticides in the blood, mainly POPs (including OCs), especially in the Alu assay [34,35]. Pesticides, and especially OPs, were shown to induce oxidative stress and reduce the activity of antioxidant enzymes in cultured cells and in vivo animal studies [42,43]. In parallel, it has been shown that carcinogenesis can result from many factors, one of which is oxidative stress, referring to a decreased antioxidant activity while reactive oxygen species (ROS) are overproduced. Oxidative stress can play a pivotal role in the pathogenesis mechanisms by disturbing normal biological functions and inducing epigenetic changes that lead to different cancer models [20,43,44,45,46]. In fact, DNA hypomethylation can be induced by the ROS through the formation of 8-hydroxy-2′-deoxyguanosine (8-OHdG), while site-specific hypermethylation can be induced by ROS via the up-regulation of expression of DNA methyltransferases (DNMTs) and of tumor suppressor gene (TSG)-promoter regions [47].

A part of the PaPOE study falls under the scope of occupational exposure to pesticide mixtures, as well as the potential effects of epigenetic alterations on the oxidative stress and cancer pathways, which have been subject to increased scientific interest in recent years. To this end, we will perform a profiling of pesticides using a biomonitoring approach in non-invasive biological matrices, including urine and hair. Questionnaire data will be used to ascertain levels of exposure over the farmworker’s lifetime. Afterwards, global DNA methylation and the methylation of specific genes will be conducted, and levels will be correlated to occupational exposure concentrations.

#### 1.1.2. Studying the Association between Maternal Exposure to Pesticides during Pregnancy and DNA Methylation Patterns in Genes Related to DNA Damage and Oxidative Stress Pathway

Women are exposed via different pathways, including inhalation, dietary ingestion through food consumption and water drinking, para-occupational exposure occurring through the introduction of pesticides into the home by household members who have had direct contact with pesticides at work, and also through residential exposure when women are manipulating pesticides in the home and/or garden [48]. Even if men might be directly exposed to pesticides, some studies concluded that women showed higher pesticide metabolite levels [49].

Furthermore, pesticides have been associated with unfamiliar measures of fetal growth. This is the case of the association between maternal urinary DAPs and the decreased fetal weight, and the length measured during mid-pregnancy among women from the Dutch cohort [50]. They also have been implicated in the development of long-term health problems in the offspring following maternal and subsequent in utero exposure. The mechanisms involved are complex, but often involve epigenetic changes which disrupt normal cell processes, leading to the development of cancers and also the dysregulation of biochemical pathways. Furthermore, it has been established that pregnant women may not always offer their babies a safe uterine environment. In fact, the placental barrier is permeable to many environmental chemicals, which might have deleterious effects on the fetus [51]. In other words, prenatal and early postnatal exposures to environmental factors such as pesticides are considered responsible for the increasing prevalence of chronic diseases. This was hypothesized as the DoHAD approach (Developmental Origins of Health and Disease), and was highlighted by epidemiological studies and also confirmed by some animal studies, such as the one targeting permethrin, for which inter-generational PERM-induced damage on progenies has been identified for the first time [52]. In this regard, epigenetic changes are presented as mediators of these long-term effects, but the findings of previous studies are still inconsistent and lack mechanistic information.

To bridge the gap, the second part of the PaPOE study aims to investigate the effect of maternal pesticides exposure on epigenetic alterations in subsequent generations and their underlying mechanisms. Repeated assessments of potential biomarkers may help to significantly advance the understanding of the impact of pesticides on DNA methylation and on oxidative balance, resulting in an increased susceptibility to develop a cancer. We will discuss the cause–consequence relations between pesticides exposure, oxidative stress, and DNA methylation.

#### 1.1.3. Understanding Trans-Generational Effects of Parental Pesticides Exposure Using Epigenetic Biomarkers

Pivotal to the DoHAD hypothesis is the synergistic dimensions of maternal and paternal environments, which can result in a parenting system impacting the offspring development [53]. Even though parent–offspring interactions occur primarily through the mother during early life, it is increasingly clear that epigenetic variations arising in the male germline are passed on to subsequent generations, thus leading to a trans-generational transmission of epigenetic susceptibility and biological phenotypes. This is why it was speculated that fathers can pass on environmentally induced effects to offspring [53,54]. However, the underlying trans-generational mechanisms remain to be fully determined.

Following form Aims 1 and 2, the third part of PaPOE thus aims to provide insight into the epigenetic mechanisms following pesticides exposure, which might have a trans-generational effect based on the maternal and paternal environments.

In contrast to most of the previously conducted research, PaPOE will simultaneously monitor different biological matrices to predict the effects of pesticides exposure at different levels (occupational, during pregnancy, and in postnatal life). Combining the biomonitoring of chemical mixtures and the identification of effect biomarkers in several human matrices and at different stages of life is an innovative and structured approach to elucidate the exposure/effect ratio and to strengthen the understanding of the mechanistic pathways that lead to a disruption in the biological functions. With annual assessment and monitoring waves, we aim to identify those protective and risk factors with the strongest predictive value for epigenetic changes and the onset and impact of oxidative stress.

## 2. Materials and Methods

### 2.1. Study Area

**Figure 1:**
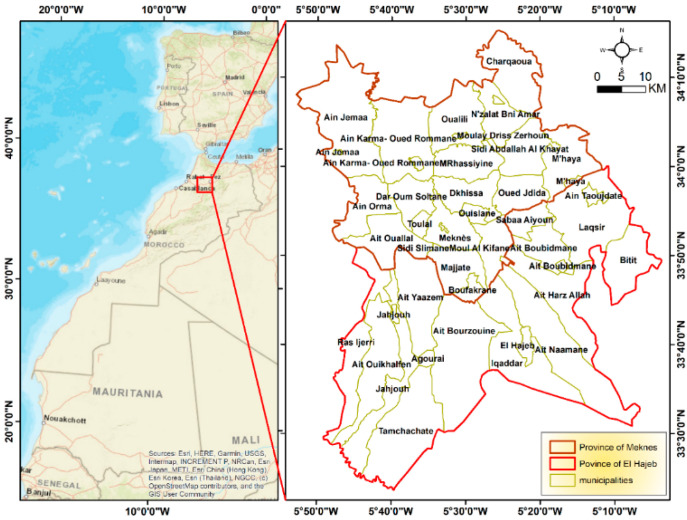
PaPOE Study area.

Meknes City (Figure 1) (34°02′00″ N, 5°00′00″ W) is a subdivision of the Fes-Meknes region according to the administrative division of 2015, extending over an area of 1786 km^2^. It is subdivided into 21 municipalities, including 15 rural towns [55]. It is well-known for its agricultural richness. Almost 143,000 ha has been cultivated from 2012–2013. The region occupies a leading position in certain crops and agricultural activities thanks to the geographical potentialities and industrial advantages it has. The main present crops are cereals, accounting for 76,500 ha of the cultivated area. Fruit trees are planted over 33,900 ha, while vegetables and oil crops cover, respectively, 17,200 ha and 2500 ha. The agricultural field is mainly male-oriented; few women work officially on the field. They can help their husbands with few tasks like pesticide application or crops transport. They are then exposed through agricultural activities, but also from exposure in the home and in public spaces. They come into contact with pesticides through washing pesticide-soaked clothes and disposing of empty containers from family members, or more indirectly, from diet.

### 2.2. Recruitment and Sampling Design

**Figure 2:**
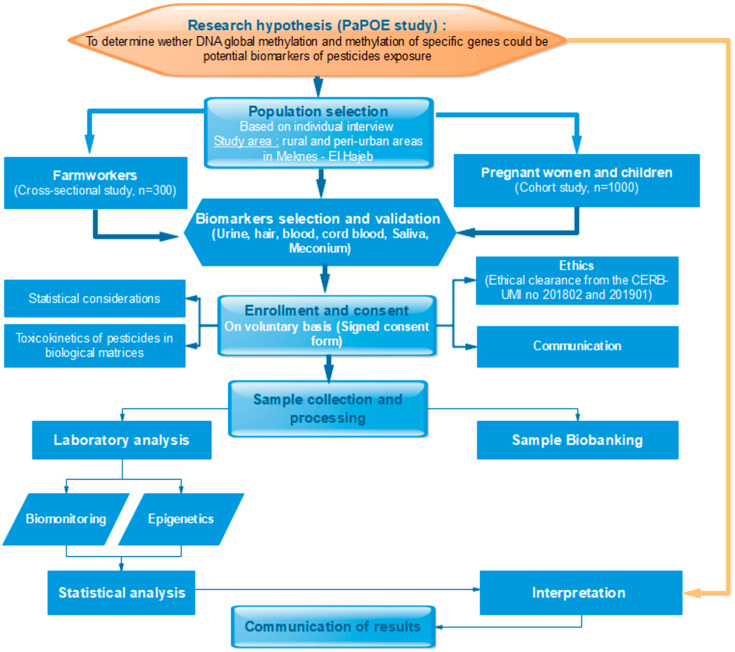
Flowchart of the PaPOE study.

The PaPOE (Figure 2) is a combination of a cross-sectional study among work-exposed men and a prospective cohort study among mother–child pairs, with a planned duration of 5 years, based in Meknes, Morocco.

We aim to recruit 300 farmworkers. Multiple visits are being conducted on the field to reach the targeted sample size. To account for seasonal variation, recruitment in each area will be spread over a one-year period. Two periods of a crop season will be surveyed: low exposure (which extends from July to December), when pesticides are used occasionally (one to two applications per trimester), and high exposure (which extends from February to June), when pesticides are used regularly on a weekly basis. The recruited group will be stratified for age and residency area (Urban, Sub-urban, Rural). The control group will consist of 100 healthy sex- and age-matched individuals living in the same area as farmworkers but without previous or current occupational exposure to pesticides, and for whom parents were not occupationally exposed. To date, 300 farmworkers have been randomly recruited within the study area.

For the cohort study, 1000 pregnant women who attend the Pagnon Public Hospital or partner clinics and care centers in Meknes and the Maternal Care Center in El Hajeb, are being recruited over a 2-year period. We will follow infants up to 2 years of age. Yearly postnatal visits will be planned at the Pagnon Hospital. According to the Moroccan Ministry of Health, the Birthing Centers in our study area (Meknes and El Hajeb, accounting for rural and urban expected births) are expected to perform 20,482 deliveries in 2020, which allows us to have 1000 eligible participants. The sampling method is non-probabilistic, according to a proportional stratified random technique of the sample, guaranteeing an equal chance to be selected for participation to all women. A proportional stratified random sample is adopted, dividing the target population into relatively homogeneous subgroups and considering the stratification variable mainly related to the ages of women between 18 and 40 years old. A number of women from each stratum is randomly selected, which corresponds to the percentage represented by the stratification variable of the target population according to geographic location. With this technique, we ensure the representation of a determined fraction of the population and allow comparisons to be made between the constituted subgroups. A total of 1000 women will be recruited at the urban and rural health centers of the prefecture of Meknes (78%) and the province of El Hajeb (22%). Out of 197 pregnant women who attended the maternity care during a 3-month period, 125 (63.45%) signed the consent form to participate.

Inclusion criteria will be, for both groups, as following:

- For farmworkers: Recruitment will target adults aged between 18 and 60 years living in the study area for at least 1 year prior to the first recruitment, and who are official or seasonal farmworkers. A control group of sex- and age-matched individuals will also be recruited at the same time, and will include healthy adults living in our study area and for whom their jobs are not linked to agriculture and do not imply the use of pesticides.
- For mother–infant pairs: Pregnant women are healthy adults aged between 18 and 40 years living in the study area for at least 1 year prior to pregnancy. Only healthy newborns will be included and followed.

Subjects will be invited to participate in the study, with adequate time given for them to consider whether they wish to participate. Patients give their consent by signing the consent form (available in French and Arabic). Then, they will be asked some questions that seek information regarding their personal information, daily life, pregnancy conditions, food intake and diet, tobacco-smoking status, health status, and medication. During the study, the response rate will be monitored (to guaranty a sufficient sample size).

### 2.3. Recruitment Procedure

Two recruitment strategies are employed in parallel, as described below:

Sample 1: Farmworkers are recruited through medical caravans organized by local associations in Meknes, in partnership with the Health Delegation of Meknes and Mohamed V Hospital, using a face-to-face recruitment method. During the caravan, the population will also get free medical care. The number of individuals has been set to 300 farmworkers and applicators. The selection of specific areas is based on the highest pesticide consumption, extensive agriculture production, and cancer rates in each zone.

Sample 2: Women are recruited during the control consultation and at delivery at Pagnon Hospital and partner clinics in Meknes and El Hajeb. The selection of subjects will be processed in the presence of a medical doctor and trained nurses and midwives. Furthermore, a gynecologist will be invited to join the medical caravan, so that pregnant women with direct contact with subjects participating in the cross-sectional study (the wives and newborns of farmworkers), or at least living in farm homes or in agricultural areas, can be included.

### 2.4. Data Collection

#### 2.4.1. Pesticides Use Assessment

Based on the fact that multiple factors can influence the carcinogenic properties of pesticides, including age, sex, individual susceptibility, amount and duration of exposure, and simultaneous use and/or exposure to other chemicals [56], a questionnaire in French (with an Arabic dialect version to support interviews) was prepared to assess work pesticides exposure. The questionnaire was divided into 3 sections that related to (a) the personal information and socio-demographic characteristics of respondents; (b) information about crop types and use of pesticides, seeking to determine the mainly used types of pesticides and how they are applied (e.g., with the use of personal protective equipment); and (c) exposure and health status related to complex exposure on the farm, such as waste, craft fumes, dust, and other activities, and to the non-farm activities and other exposures that may affect disease risks, such as diet, alcohol consumption, smoking history, medical conditions, and family histories of cancer, to screen for risks.

To account for eventual interaction, we considered the regional background levels of fine particulate matter (particles with a diameter between 2.5 μm and 10 μm) and nitrogen dioxide for all regions in our study area by using data from the recent research work of our team [57,58,59]. On that basis, we selected 2 regions as the polluted and unpolluted areas of interest. Healthy subjects in the two sub-studies living in the selected polluted and non-polluted regions are being enrolled in this study.

#### 2.4.2. Dietary Intake

Dietary intake represents a major source of pesticides exposure for infants and children. Some studies examined dietary exposures using biomonitoring and showed a correlation between some food intake and pesticides levels in biological matrices [60,61].

In our study, trained research staff will interview women before delivery, based on a standardized maternal health history questionnaire and an adapted food frequency questionnaire inspired by the FFQ and developed by [62], taking into account the Moroccan and Mediterranean diets.

#### 2.4.3. Sampling Procedure

Urine samples from participants will be collected in a polyethylene bottle and centrifuged at 1500 rpm for 15 min, and the supernatant will be stored at −80 °C for possible future analysis of pesticides metabolites. Pesticides and their metabolites will be extracted from urine, following an optimized method based on methodologies suggested by [63,64]. Briefly, non-polar and polar pesticides will be simultaneously extracted from urine samples by a solid phase extraction (SPE) procedure, using C18 cartridges as sorbent. A 5-mL aliquot of urine, diluted 1:1 (*v*/*v*) with MilliQ water, with an added volume of the internal standard, will be loaded onto a Sep-Pak^®^ C18 cartridge that has been preconditioned with 4 mL dichloromethane and 4 mL of MilliQ water. The sample will be eluted and then dried under a speed vacuum concentrator (Thermo Electron Corporation, Waltham MA, USA). The residue will be dissolved in 2 mL ethyl acetate. A total of 1 mL is injected to the GC-MS Agilent Technologies GC system (6890A) equipped with an RXi-5MS capillary column (60 m, 320 mm, 1.00 mm; Agilent Technologies, Santa Clara CA, USA). The other 1 mL is, again, dried and dissolved in 1 mL aqueous solution of methanol 1:1 (0.01% formic acid) to be analyzed using a Waters Acquity Ultra-Performance Liquid Chromatograph equipped with a Xevo TQ-XS Tandem Quadrupole Mass Spectrometer (Waters, Guyancourt, France).

Blood samples will be collected from volunteers in convenient collection tubes, taking into consideration all the endpoints for which these tubes will serve. It will concern farmers’ blood, maternal blood, and cord blood. For this purpose, blood samples taken from the mother are collected just before delivery, as women are allowed to enter the study during any stage of pregnancy. Next to that, cord blood is collected from each participant at birth, as it is considered a proxy for prenatal exposure and collection is non-invasive for both mother and child. Whole-blood and plasma samples will be kept frozen at −80 °C for possible future analysis.

Hair samples will be collected from the posterior vertex region of the head, close to the scalp, as recommended by the Society of Hair Testing guidelines [65]. The minimal amount of hair sampled for analysis needs to be at least of 70 mg, and hair strains will be of pencil-width thickness. Each hair sample will be placed in a foil in a sealed and labeled plastic bag for future decontamination and analysis for the selected metabolites of pesticides using LC-MS and GC-MS, based on the methodology developed by [66].

Meconium samples will be collected from the 2 stools of the baby during the 18 h following the birth, and then stored at 4 °C. It is formed by, and accumulates in, the fetus from the 12th week of gestation until birth, and is a repository of the xenobiotics that the fetus has been exposed to throughout this period. Hence, meconium provides a wide window of fetal exposure to environmental agents and, therefore, is an ideal matrix to analyze. Before a solid–liquid extraction, the meconium sample will be dried and ground. The extract will then be purified using a weak anion mixed-mode polymeric sorbent in a solid-phase extraction. The sample will be analyzed with LC/MS, following the method developed by [67].

Finally, at 12 months, a buccal cells sample of the child is collected to determine the status of DNA methylation. In children (6 months–11 years), buccal sampling is much more tolerable than blood sampling, hence the relevance of our choice.

Table 1 shows the correspondent samples that will be drawn from each participant and the analysis to be performed.

### 2.5. Human Biomonitoring

#### 2.5.1. Quality Control

Blanks will be used for measuring existing contamination or bias in the laboratory environment, including the material and solvents. Blanks will then be analyzed for every set of 20 biological samples. Three blanks fortified with the analytes will be prepared at low and high concentrations. Calibration straight lines will be prepared.

Biomarker values will be reported if they are above a quantifiable level, as determined in the laboratory. Values below the detection limit (LOD) or quantification limit (LOQ) will be replaced by LOD/2 or LOQ/2. For the purpose of quality control, triplicate samples will be included.

#### 2.5.2. Exposure Biomarkers

For all collected samples of urine, hair, and meconium, a quantification of different chemical classes of pesticides (organochlorides, organophosphates, pyrethroids, neonicotinoids, carbamates, auxins, and others) and their metabolites will be processed using HPLC/MS and GC/MS.

The selection of these compounds was done according to three criteria: (i) high diversity of chemical families for the parent compounds, (ii) wide ranges of variation of the values of environmental and molecular parameters, and (iii) use of pesticides in the study area, following a survey conducted in 2008 and 2016. The list of pesticides included chemicals classically investigated in humans (e.g., OCs, OPs, and pyrethroids) in order to allow for a comparison with data obtained from other population-based studies.

The inclusion of pesticides into the study is restricted to those for which validated analytical methods are available to the project team to analyze for the associated urinary metabolites. As further validated methods become available during the study, the list of pesticides for inclusion may be expanded.

According to an EFSA supporting publication, the most-studied herbicides (in order) were shown to be: 2,4-D > atrazine > glyphosate. Similarly, the most studied insecticides (in order) were: chlorpyrifos > permethrin > cypermethrin = deltamethrin > malathion, and the most studied fungicides were: captan > mancozeb [8]. We also based our selection of potentially carcinogenic chemicals on the outcome of hazard evaluations by the International Agency for Research on Cancer (IARC): (i) known human carcinogens (Group 1) included Lindane, pentachlorophenol (PCP), cadmium, chromium, and arsenic; (ii) probable human carcinogens (Group 2A) included glyphosate, dichlorodiphenyltrichloroethane (DDT), dieldrin, malathion, diazinon, and OPs; and (iii) possible human carcinogens (Group 2B) included parathion, chlordane, Mirex, hexachlorobenzene (HCB), di(2-ethylhexyl) phthalate (DEHP), perfluorooctanoic acid (PFOA), and lead [68,69,70].

Metabolites of organophosphate pesticides (dimethyl phosphate (DMP), dimethyl thiophosphate (DMTP), diethyl phosphate (DEP), and diethyl thiophosphate (DETP)) are determined in urine and hair samples using derivatization and gas chromatography mass-spectrometry detection, following the method used by Hardt and Angerer [71].

Metabolites of glyphosate, including aminomethylphosphonic acid (AMPA), hydroxymethylphosphonic acid (HMPA), and glufosinate, are determined in urine and hair samples using FMOC derivatization and LC-MS detection, following an adapted selected method. For the extraction, commercial molecularly imprinted polymers AFFINIMIP^®^SPE, glyphosate, and AMPA are used. They are selective solid-phase extraction cartridges that selectively clean and concentrate glyphosate, AMPA, and glufosinate prior to analysis by HPLC [72]. For derivatization, the reaction was accelerated by a 20-min incubation at 50 °C. The quench reaction uses phosphoric acid. Any precipitate is dissolved using methanol.

To adjust for urine dilution, creatinine concentrations are measured using the Jaffe reaction using a commercial kit (Roche Cobas, Meylan, France) and pesticide concentrations are corrected.

Serum pesticides will be expressed by lipid weight. Serum total cholesterol and triglycerides will be measured colorimetrically using commercial kits (Roche Cobas, Meylan, France).

#### 2.5.3. Method Validation

For each analytical procedure, matrix-matched calibration curves are obtained by supplementing a blank with the appropriate standard solution in order to cover different concentration ranges, according to the analyzed matrix. Several hair and urine samples obtained from healthy volunteers without occupational exposure to pesticides are therefore analyzed, and the samples containing the lowest pesticide concentration are used for the method validation.

### 2.6. Assessment of the Biological Activities

#### 2.6.1. Paraoxonase 1 Activity

Paraoxonase (PON) is a multifunctional enzyme involved in oxidative stress that detoxifies several Ops, such as chlorpyrifos, parathion, and diazinon. A low level of PON1 activity may result in an elevated sensitivity of newborns to OP toxicity [73]. Several polymorphisms in the PON1 gene affect the function, production, and efficiency of PON in the body; recent studies have focused on two alleles: PON1_-Q192_ and PON1_–108_ [74,75].

PON1 catalytic activity will be measured using paraoxon (1.5 mM) as substrate, freshly prepared in 50 mM glycine buffer pH 10, containing 1mM calcium chloride, and incubated at 25 °C with 50 μL of serum. The liberation of p-nitrophenol upon enzymatic hydrolysis will be detected by a spectrophotometer measured at 405 nm. Results will be expressed as U/L.

#### 2.6.2. Cholinesterase Activity

Evidence indicated that OPs exert their toxicity through the inhibition of acetylcholinesterase (AChE) [43]. In fact, AChE has non-cholinergic functions contributing to the control of cell proliferation and apoptosis [76], which means that OPs may increase the carcinogenic risk through the inhibition of a tumor suppressor (AChE). The detection of AChE activity can thus provide an idea about the exposure to these chemicals and their carcinogenic effects.

Plasma and erythrocyte acetylcholinesterase (BChE and AChE, respectively) are determined within 4 min at ambient temperatures of 10 °C to 50 °C, using a commercial cholinesterase kit (ChE rapid test IVD) from Roche Cobas (Meylan, France). The reaction mixture is incubated for 5 min at 25 °C. A total of 170 µL of defrosted whole blood, diluted 1:100 with distilled water, is added. The thiocholine produced in the presence of the highly reactive DTNB ion generates a yellow color, which is quantified using a spectrophotometer at a wavelength of 410 nm. Activity is expressed as μmol/min/g hemoglobin. Changes in AChE and BChE activity are quantified before and after exposure to OP pesticides.

#### 2.6.3. Antioxidant Enzyme Activity Detection

Superoxide dismutase (SOD) helps in the defense against oxygen radicals [77]. For its measurement, the colored formazan, formed from the reaction between superoxide radicals—produced from the xanthine–xanthine oxidase system—and radical iodonitrotetrazolium (INT), is measured over a 3-min period at a wavelength of 505 nm. The reaction is inhibited in a manner dependent on the CuZn-SOD activity in the medium. The level of inhibition is measured and the SOD level in U/g is determined [78].

Malondialdehyde (MDA) can react with nitrogen bases and might thus be mutagenic and carcinogenic [45]. Incubation of MDA with thiobarbituric acid at 95 °C and pH 3.4 forms a pink complex which can be measured by spectrophotometry at 532 nm. The level of this complex is expressed in nmol/mL of serum malondialdehyde.

Glutathione (GSH), oxidized to glutathione disulfide (GSSH), is involved in clearing ROS. Their levels are determined using LC/MS after sample stabilization with N-ethyl maleimide (NEM) at 400 Mm. GSH and GSSG concentrations in blood are analyzed using the previously published method by [79]. Briefly, frozen blood samples were homogenized on ice in cold 40 mM N-ethylmaleimide (*v*/*v*), to prevent the rapid oxidation of GSH, and centrifuged at 14,000× *g* for 15 minutes at 4 °C. The supernatant is then transferred to a new tube, where a 5% metaphosphoric acid is added to remove the proteins. The mix is again centrifuged at 14,000× *g* for 15 minutes at 4 °C. The supernatant is stored at −80 °C until measurement. Levels of reduced and oxidized forms of glutathione are performed using ultra-pressure liquid chromatography (UPLC), in combination with tandem mass-spectrometry (MS-MS). The LC/MS-MS analysis will be conducted on a Waters^®^ Acquity UPLC^TM^, coupled to a Waters^®^ Micromass Quattro Premier^TM^ Mass Spectrometer using electro spray ionization (ESI). Data will be further normalized to protein content measured using the Bradford method and a bovine serum albumin as the standard. The ratio of total glutathione to glutathione disulfide is then calculated.

8-OHdG is a marker of DNA repair. A commercial kit (Epiquick 8-OHdG DNA damage quantification direct kit) (EpiGentek, Farmingdale NY, USA) is used directly on the DNA extract to quantify 8-OHdG using spectrophotometry.

### 2.7. Epigenetic Biomarkers

There is growing scientific interest in describing the contribution of epigenetic events in the initiation, promotion, and progression of different types of cancers, mainly through the silencing of tumor suppressor genes and/or the activation of proto-oncogenes. DNA methylation is, to date, the best-known epigenetic mechanism in the human genome. Methylation consists in the addition of a methyl group at the carbon 5 position of the cytosine ring, resulting in the 5-methylcytosine. It mainly occurs in cytosines of CpG islands [62,80]. Altered DNA methylation patterns are hallmarks of human cancers. Hypomethylated sequences can abnormally activate genes that are normally repressed, while hypermethylation induces the silencing of interesting genes such as tumor suppressors. These events can be detected in blood and saliva, and can thus serve as predictable biomarkers for human cancers [81]. Pivotal to this emerging idea was the lack of knowledge regarding the effects of pesticides on DNA methylation.

The elevated cancer risk following exposure to pesticides indicates a gap in the current knowledge of pesticide carcinogenicity, and provides evidence that pesticides may cause cancer through alternative mechanisms, such as epigenetic changes [1].

In this context, the PaPOE study will focus on global DNAm and critical target loci within genes whose expression is likely to be affected by pesticides exposure and which are commonly found to be aberrantly methylated in cancers.

#### Global Methylation and Hydroxymethylation

Genomic DNA will be extracted from buccal cells and whole blood using the QIAamp DNA Mini Kit (Qiagen, Hilden, Germany), according to the manufacturer’s protocol. The quantity and purity of DNA will be determined by a NanoDrop 2000c spectrophotometer (Thermo Fisher Scientific, Waltham, MA, USA).

Global genomic DNA methylation and hydroxymethylation of cytosine and adenosine nucleotides will be quantified and compared in human DNA for each sample using ultra-pressure liquid-chromatography, combined with tandem mass-spectrometry, as previously described by [82]. Briefly, 500 ng are used as input DNA. Internal standards are 15N3 and 8-OHdG. Global DNA methylation will be assessed by quantifying 5-methyl-2′-deoxycytidine (5mDC), 5-hydroxymethyl-2′-deoxycytidine (5hmDC), 5-formyl-2′-deoxycytidine (5fDC), 5-caroxy-2′-deoxycytidine (5caDC), and 2′-deoxycytidine (DC) and N6-methadine (6mA) using UPLC/MS-MS, conducted on a Waters^®^ Acquity UPLCTM, coupled to a Waters^®^ Micromass Quattro Premier TM Mass Spectrometer using electro spray ionization (ESI).

Genomic DNA (200 ng) will be bisulfite, converted using the EZ-96 DNA Methylation-GoldTM Kit (#D5008, Zymo Research, Irvine CA, USA), and then amplified by PCR in a total volume of 25 mL containing 0.2 mM of primers and 2× Qiagen PyroMark PCR Master Mix (#978,703, Qiagen). Pyrosequencing will be performed using Pyro Gold reagents (#970,802, Qiagen) on the PyroMark Q24 instrument (Qiagen), following the manufacturer’s instructions. Pyrosequencing results will be analyzed using the PyroMark analysis 2.0.7 software (Qiagen, Hilden, Germany).

Long interspersed nucleotide elements (LINE-1) and the AluX (Alu) methylation levels will be assessed using PCR-pyrosequencing of bisulfite-treated DNA. Details of the PCR-pyrosequencing assays to be used in the current study are described by [62].

The degree of methylation will be expressed as the percentage of methylated cytosines divided by the sum of methylated and unmethylated cytosines (% 5mC).

### 2.8. Sample Size and Data Analysis

The sample size for global methylation outcomes is determined assuming a 0.2 standard deviation from the median methylation concentrations in the exposed group compared to the control group. This corresponds to observed differences found in studies investigating the impact of pesticides exposure on epigenetics outcomes, using metabolites as the method to assess for pesticide exposure in the United States and in Belgium [83,84]. A sample size of 300 is judged to be adequate, with a power of 80% and a 5% level of significance for farmworkers.

Sample size calculations for epigenetic outcomes among pregnant women and newborns are determined using findings from a previous cohort study conducted by our team [83], which showed differences in the same epigenetic outcomes as in this study. The epigenetic outcome requiring the highest sample size to show a significant difference is for global DNA methylation (one of the epigenetic outcomes to be measured), for which a sample of 498 pairs is required to ensure sufficient power in the study.

The mean (standard deviation) and the frequency distribution will be calculated to describe the demographic and socio-environmental characteristics of the study population. When appropriate, median and interquartile ranges will be presented.

Disease and epigenetic modifications are the outcome of many component characters; thus, assessing the extent of the contribution of each factor is important in designing an effective preventive and curative approach. A path coefficient analysis will be carried-out to determine the direct and indirect effects of each independent variable on the dependent variables, i.e., to determine the direct and indirect pathway effects of, first, sociodemographic, lifestyle, and knowledge of pesticide practices and perceptions among farmers; and second, to determine the link between these pathway effects and pesticide concentrations, and then to epigenetic outcome. Path coefficient analysis is useful since it reveals the nature of the cause-and-effect relationships of exposure–outcome relationships.

For statistical comparison, the unit of pesticide concentrations in urine, hair, and meconium will be expressed in μg/mL, to be consistent with the concentration unit for blood. The frequency of pesticide exposure will be compared among the four matrices via the Cochran Q test. For very low frequency, an exact test will be used. The concentrations of pesticides, presented as median and interquartile range, will be compared via the Friedman test.

To investigate to what extent the results obtained from hair and urine analyses may help to accurately categorize individuals according to their level of exposure, a reverse-classification analysis (RCA) will be conducted, based on the approach described for humans in [85]. The nonparametric Mann–Whitney U test will be used to compare levels of biomarkers of oxidative stress between farmworkers and controls. The non-parametric Wilcoxon test will be used to test for significant differences between the biomarkers studied for both exposure periods (high and low exposure to pesticides). Since data for biomarkers of oxidative stress will be available for each individual of the cross-sectional study at two different time points, a linear mixed-model analysis will be used to evaluate the influence of pesticide exposure on repeated measures of biomarker levels.

Sex, maternal age (continuous), pregnancy body mass index (BMI, continuous), current smoking status (no, if nonsmoking with no passive exposure at home; yes, if actively smoking), education (no education, <high school degree, or >undergraduate degree) will be included as covariates in all logistic regression models.

Furthermore, sensitivity analyses will be performed to explore whether mothers’ pregnancy medical conditions might confound the relationship between urinary and hair concentrations and birth outcomes (birth weight, cord blood DNA methylation). New-born sex and gestational age will be considered as an effect modifier.

## 3. Results and Discussion

The findings are expected to reveal significant changes in DNA methylation related to pesticides exposure. This study will place a strong focus on early molecular mechanisms to bridge the gap between pesticides exposure and disease and to identify cancer biomarkers for the detection of inheritable changes. It illustrates the potential of using epigenetic markers to forge links in the causal chain for pesticides exposure and cancer risk. Statistical analysis will be used to support the answer to this hypothesis.

The combination of biological specimens and questionnaire data on lifestyle and exposures provides unique possibilities for studying the effects of many factors of relevance for pregnancy outcomes and trans-generational effects. In order to get more insight into the biological mechanisms triggered by gene–environment interactions and disease, the working range of our study covers both environmental and occupational exposure.

The multi-residue analysis is used and thus considers the independent but also synergistic/cocktail effects of the targeted pesticides, where the toxicity of pyrethroids and triazines are potentiated by OPs, which themselves can be potentiated by previous exposure to OCs, and where carbamates and pyrethroids have a synergic effect [86].

The design of our study allows us to give an approximate estimation of pollutant levels in Moroccan adults. It encompasses results from a wide cross-generational sample, allowing for a better exploration of the mechanisms involved throughout the life-course, while providing an insight into the particular susceptibility of little children. The ambitious design is suitable to be made nation-wide, thus supporting efficient public health measures related to the surveillance of the selected pollutants and an adoption of a biomonitoring program at the national or continental level.

In this sense, the PaPOE study is similar to the ENVIRONAGE project conducted among the Belgian population, supporting the hypothesis that environmental exposure may conduct to unhealthy development by studying the exposure during the most vulnerable stages in life, including the fetal window [87]. The PaPOE, in its part related to the launch of a birth cohort study, is inspired by the MANOE study in which the aim was to determine the effects of dietary methyl-group intake during pregnancy on the DNA methylation pattern of mother and child [62].

Through several Ph.D. and post-doc projects, the PaPOE has the ambition to define levels of environmental pollution in water and air and to monitor the internal human dose using the biomonitoring approach, with the aim of investigating the impacts of pesticides and other assimilated environmental pollutants’ exposure on oxidative stress, preterm birth, hypovitaminosis D, and cancer.

## 4. Strengths and Limitations of This Study

PaPOE (Parental Pesticide and Offspring Epigenome Study) will be, in Morocco, the first dedicated occupational and longitudinal study yet undertaken to investigate pesticides exposure and their effects on human health.

The wealth of occupational and longitudinal data, in addition to biospecimens collected in the framework of our study, will enable an unparalleled investigation of the factors contributing to adverse effects of pesticides exposure and early-life health and disease.

The major assets of the PaPOE approach are its detailed occupational and environmental determinants, in combination with the longitudinal health data registered in general practice, in addition to the linkage to disease and mortality registries and self-reported health.

Self-reported information is highly supported by biomonitoring measures, which allow for a better characterization of the exposure.

Nutrition and other sources of exposure (air pollution, water pollution) are taken into account, allowing for a better understanding of the exposure.

Pesticide mixtures are a big concern. We included the number of pesticides based on their extensive use in the study area and/or their suspected carcinogenic effect. The multi-residue analysis is preferred and will consider the synergistic/cocktail effects of the targeted pesticides. This facilitates comparisons with previous studies, but also adds to the body of knowledge on less frequently studied compounds. Studies which have assessed such a broad range of environmental chemicals in a variety of samples from a prospective mother–child cohort have, thus far, been few. We will, furthermore, aim to look further than the compound-by-compound approach, and to look at chemical mixtures, amongst others, by studying the biomarkers of the effects.

Genetic variation is not considered unless we are provided the chance to recruit some twin pregnancies.

A focus on participants from the Meknes region (Morocco) may limit the extrapolation of some findings to the Moroccan population.

Efforts are made to keep drop-out rates, amongst others, as low as possible by regularly sharing the results of the study and translating these to daily life applications. However, due to the duration of the study for each participant, loss to follow-up is inevitable.

The lack of a significant sample of farmworkers/pregnant women couples may limit the possibility of providing a conclusive explanation of the contribution of each parental exposure to the offspring profile.

It is not intended to provide participants with individual results, due to the novelty of the measurement methodology and the difficulty of comparing the participating individual urine, blood, and saliva results with any legal exposure limits. However, the collective results of these sampling matrices will be published in the study report and in various peer-reviewed publications.

The health system in Morocco does not allow for a good follow-up of women in their first trimester of pregnancy. Only questionnaires and medical records will be used to trace the exposure history during the early months of pregnancy.

## 5. Conclusions

The PaPOE study will help to track the full continuum from occupational and maternal pesticides exposure to offspring epigenotypes to later phenotypes. The use of the human biomonitoring approach will contribute to the highlighting of personal living and/or working conditions and protective practices, taking into account individual differences. In our case, the use of several biological matrices will also improve the health hazard identification and give a more concrete picture of the exposure.

As it is increasingly clear that epigenetic alterations, including abnormal DNA methylation, are involved in carcinogenesis, devoting more energy to explore this epigenetic component should bring new insights to better explain cancer diversity.

This protocol presents an integrative methodological framework presenting the major steps in the source-to-effect continuum. It is a promising approach that elucidates the causal link between the exposure to chemical agents and health outcomes, using biomonitoring and biochemical and molecular effect biomarkers.

Occupationally or environmentally, humans are blindly exposed to different xenobiotics at a time. As pesticides are classified into several chemical families and are used simultaneously or gradually on the field, synergistic and/or potentiating responses are likely to occur. Therefore, studying the occurrence and the impacts of this interaction, even if complex and challenging, has never been more critical. Given that fact, our framework considers the investigation into the exposure to chemical mixtures by using a multi-residue analysis and nonspecific biomarkers of oxidative stress in different body fluids.

Our ambition is to extend follow-ups with our participants throughout childhood and, hopefully, adolescence, to further study the long-term health effects of environmental exposures. We are now in the phase of prospective follow-ups, with the aim of continuing this for as long as possible (12+ years), pending future funding.

With the objective of addressing knowledge gaps and promoting innovative approaches, the PaPOE study will provide better evidence of the actual exposure of the Moroccan population to pesticides, and the possible early health effects. The ultimate aim is to advance human biomonitoring in Morocco and Africa and to support policy making.

Findings will be disseminated through scientific conferences and peer-reviewed journals, and through newsletters and the project website to participants, stakeholders, and the wider public. Based on the used approaches, our findings will allow for more individualized measures to prevent exposure by inciting individual behavioral changes.

By introducing the study protocol, we hope to stimulate other research groups to replicate and extend the design to ensure comparability between studies and more valid and generalizable conclusions about the biomonitoring results and health impacts of implementing environmental cohort studies in the south Mediterranean shore.

## Figures and Tables

**Table 1:** Data and specimens collected.

Data Sources	Description	Purpose
Environmental and occupational exposure questionnaire	Environmental exposures, source of drinking water, organic eating behaviors, residence history, occupation.	Gather socio-demographic data and understand the occupational exposure of farmers.
Maternal health history questionnaire	Personal medical history, reproductive history, pregnancy status, family history of cancer, demographic data.	Gather socio-demographic data and identify any health risks on pregnancy.
Food Frequency questionnaire	Summary of dietary intake frequencies over the previous year.	Usual food consumption, including portion sizes, during the previous 12 months. Nutrient and food group intake estimates.
Dietary recalls	Complete report of all food, drink, and supplements consumed the previous day (3 recalls were requested).	Validate data reported on the FFQ and reduce the risk of bias.
**Specimens collected**	**Description**	**Purpose**
Urine	First-void urine samples	Biomonitoring of pesticides metabolites
Blood	Peripheral blood, separated into serum for biochemical analysis and biomonitoring, and whole blood for DNA extraction. It includes blood from farmers, maternal blood prior to and during delivery, and cord blood.	Biomonitoring. Complementary biochemical analysis. Oxidative stress biomarkers quantification. DNA Methylation.
Hair	Optional sample	Biomonitoring of pesticides.
Buccal cells	Optional sample, collected from infants in the cohort study.	DNA extraction. Biomarkers identification. Epigenetics.
Meconium	2 stools of the baby.	Biomonitoring of pesticides.
